# “It’s Not Good for the Animals, but I Think It Should Be Done”—Using Focus Group Interviews to Explore Adolescent Views on Animal Experimentation

**DOI:** 10.3390/ani12172233

**Published:** 2022-08-30

**Authors:** Sonja M. Enzinger, Christian Dürnberger

**Affiliations:** 1 Institute for Secondary Teacher Education, University College of Teacher Education Styria, Hasnerplatz 12, 8010 Graz, Austria; 2 Unit of Ethics and Human-Animal-Studies, Messerli Research Institute, University of Veterinary Medicine Vienna, Veterinärplatz 1, 1210 Vienna, Austria

**Keywords:** animal testing, animal ethics, animal welfare, human-animal relation, attitudes, public opinion, youth research, focus groups, qualitative research

## Abstract

**Simple Summary:**

There are comparatively many studies that explore how adults judge animal experiments. But how do young people think about this topic? Our group interviews conducted with Austrian teenagers showed that the participants assessed animal tests more positively than we had expected. The teenagers evaluated animal experiments mainly based on the following criteria: the relevance of research, the extent of animal suffering, and the existence of alternatives. All groups found positive aspects for animal experiments and identified acceptable animal experiments among the examples discussed. Particularly with regard to the approval of animal experiments, a key consideration was the extent to which the research is relevant to humans.

**Abstract:**

The present study focused on an in-depth analysis of adolescents’ (aged 15–16) attitudes towards animal experimentation. Focus group interviews were conducted to gain a deeper understanding regarding the ethical considerations of this age group. The data were analyzed using a qualitative content analysis. All participants considered their own knowledge about the whole topic as low. Our results show that adolescents in the study had considerably more positive attitudes toward animal experimentation than the literature had suggested. All groups identified positive aspects of animal experimentation and accepted at least one scenario of animal experimentation. Most of the groups rated half of the examples presented as acceptable. The participants tended to make specific assessments in view of a concrete scenario and seemed to form their positions anew. In their discussion, students focused mainly on the following criteria: the relevance of research, the extent of animal suffering, and the existence of alternatives. Generally, we hypothesize that the focus group discussions took place largely within the framework of anthropocentric ethics.

## 1. Introduction

Animal experimentation is a research field which has been partly critically observed and discussed in society. For example, in 2017 the University of Tübingen stopped their animal experiments on non-human primates because of massive protests in the population [1,2]. In Switzerland, there was an (unsuccessful) popular initiative in February 2022 for a complete ban on animal testing [3]. However, even if sections of the society have a critical view of animal experiments, their great importance for the development of medications and treatment methods has been repeatedly emphasized by experts [4]. Most recently, for example, this happened in connection with the management of the COVID-19 pandemic [5].

Moreover, the public understanding or acceptance of science is also reflected in its regulations as well as the funding of the research [6]. Above all, the term “public understanding” also includes that individuals gain an understanding of the extent to which science affects themselves and the society they are living in [7].

In general, it can be considered important within a democratic society to explore existing public perceptions of science and identify possible knowledge gaps or misunderstandings. After all, research on knowledge, attitudes, and perceptions about animal experimentation could foster the communication between science and the public. However, it is known that there are gaps in the general population’s knowledge of animal experimentation as, for instance, an exemplary study by Ipsos MORI in 2018 in the UK showed: 64% of the population did not feel well informed about animal research; the government’s work on the 3Rs (refine, reduce, replace) were largely unknown (by about two third of the respondents); there was still the misconception that animal testing is permissible in the cosmetics industry (38%) [8].

According to the literature there are a number of factors which influence attitudes on animal experimentation. These are: factors which are related to the instruments (e.g., wording of the questions, type of research, level of harm, availability of alternatives), factors which are related to the animals (e.g., species, sentience, genetic modification), and factors which are related to the respondents (e.g., age, gender, area of living, education, pet ownership) [9,10].

However, many such studies examining society’s attitudes toward animal experimentation use questionnaires as a method. Questionnaires can give an interesting overview of the general attitudes present in the population. Nevertheless, the use of questionnaires can frequently not provide deeper insights into ethical considerations, especially since they often investigate the participants’ general approval or disapproval of research involving animals while not addressing the underlying reasoning.

Against this background, Lund and colleagues analyzed the attitude formation of laypersons in focus group interviews. This study was able to show, among other things, that even laypersons with not much prior knowledge of animal experimentation have no difficulty forming their points of view. For the evaluation of animal experiments, according to the study, the central considerations were the perceived human benefits and animal costs. Apart from the respondents who were described as general approvers and disapprovers, the study also identified so-called “reserved” participants who had “highly elastic attitudes”, and who formed their position anew with each animal experiment that was discussed [11].

One aspect has not been addressed so far: the age of the respondents. Most studies on the acceptability of animal testing focus on adults. If young people’s perceptions of animal experimentation are surveyed, this is usually done quantitatively or their short answers are evaluated [12,13,14]. However, this (young) age group is especially important because they represent the future decision makers in society. Therefore, most science curricula in Europe do not only refer to scientific knowledge, but instead explicitly include the ethical considerations and expect teachers to embed scientific knowledge in social and cultural occurrences [15]. In the context of science education, there is also an opportunity to refer to present conceptions and knowledge gaps of adolescents regarding animal experimentation, and thus promote an informed society.

Against the background of what has been said so far, this study focuses on the perspectives of adolescents on specific examples of animal experimentation, using focus group interviews as a method. It addresses two overarching research questions: (1) What prior knowledge do adolescents have about animal experimentation? (2) Based on which criteria, do adolescents valuate animal experiments?

## 2. Materials and Methods

The study took place in June 2019 in four different upper secondary schools in Austria. Three of the schools were from an urban area in Styria and one from a rural area. All of the students had an age of 15–16 years and attended the 9th or 10th grade, respectively. Four focus group interviews were conducted with 5–6 participants each (N = 21). The students of each focus group attended the same class and knew each other before the study. The focus groups took place in a room in the student’s school during their normal school time. In the class, a teacher of the students asked if anyone was willing to participate in the focus group interviews. In case that more than six students wanted to participate, a maximum of six persons were randomly chosen. Before the focus groups took place, the approval for the study was given by the school authority (Bildungsdirektion Steiermark), which is the responsible institution for research in schools in the federal state. All school directors and parents of the participating students received written information on the study and gave their written consent.

Since the central goal of this exploratory study was to examine adolescents’ perspectives on animal testing in more detail, focus group interviews were conducted. This research method is particularly suitable for gaining in-depth knowledge about a specific research area. Through the initiated discussions between the participants, a whole variety of views can be collected [16]. Krueger and Casey describe the characteristics of focus group interviews as follows: “*(1) a small group of people, who (2) possess certain characteristics, (3) provide qualitative data (4) in a focused discussion (5) to help understand the topic of interest.*” [17]. For conducting the focus group interviews we followed general accepted rules for focus group interviews [18]. The first author [researcher SME] acted as the facilitator of the focus group interviews and guided the participants through the session. The facilitator and the participants did not know each other before the study. A facilitator is important to keep the discussion running and to ensure that all participants get the chance to speak and depict their views [18]. We prepared a guideline for the procedure of the focus group interviews. Before we conducted the study, we organized a pre-study with a test group which we did not include in the analysis. We used this pre-study to test the duration of the focus group interviews, to familiarize ourselves with the guidelines, and to check the understandability of the questions and tasks. The focus group interviews started with an introduction in which the facilitator welcomed the participants and began with some warm-up questions. The participants had the possibility to introduce themselves and to talk about their personal relationship with animals and their views on animal usage in general. After this warm-up, the facilitator led the discussion to animal experimentation as a specific example of animal use. The participants were asked to first describe their knowledge of animal experimentation and to mention possible examples. Afterwards the participants received four specific examples of animal experimentation and were asked to evaluate them individually. Three of the given examples focused on medical research and one on cosmetics. The first example focused on cancer research, with the aim of finding new therapeutic methods against cancer. Examples 2 and 3 focused on the development of new drugs against migraine and obesity. Example 4 focused on cosmetic research with the testing of a new deodorant [11]. Although animal experimentation for cosmetical products is forbidden in the European Union, we added this example since our investigation concerned the general ethical assessment, and not the current legal situation in specific countries. The examples contained the following information: species used (mice, rats, rabbits, pigs) and expected pain for the animals (pain level: no to strong), aim of the research, and a short description of the method. Afterwards the participants discussed the examples in the group and explained their views. The four examples on animal experimentation were already used in a previous study of Lund and colleagues [11] and were adapted slightly, based on the feedback we received from the students after the pre-study. We translated the examples to German and changed, for example, terms which were difficult to understand for the students and replaced them with easier descriptions. We have also restructured the information of the examples to better clarify the process of the experiments. After a general discussion of the four examples of animal experimentation, the groups were asked to decide if they would approve them. Finally, the students were asked how they viewed a complete prohibition of animal experimentation. All the focus group interviews were audio and video recorded.

For the analysis we transcribed the data verbatim and applied the qualitative content analysis of Mayring [19] using the online tool, Qcamap (https://www.qcamap.org/ui/en/home, accessed on 28 June 2022). We applied an inductive analysis technique where the category system is developed from the transcript. Before the analysis started the coders had to describe the selection criteria for new categories. Afterwards, one coder (researcher SME) went through the text, line by line, and searched for new categories. During this coding process, coding rules were developed if they were necessary. After 50% of the material was categorised, the categories were revised by both authors (SME and CD). For this process the second coder (researcher CD) independently analysed the same material with the already developed codes and the results of both coders were used to discuss and refine the categories and develop further rules for categorisation. During the further analysis of the data material, there were several feedback loops between the coders to discuss new categories. Once no new categories were found, all data were analysed by both coders to calculate the interrater reliability (IRR). We measured the IRR using Krippendorff’s Alpha [20]. After the calculation of the IRR, we resolved distinctions in the analysis of the two coders by discussion to receive the final percentage for each category.

## 3. Results

### 3.1. Knowledge and General Attitudes on Animal Experimentation

All 21 students said that they did not have much prior knowledge on animal experimentation as it is rarely a topic in school and not discussed in their families. As we did not receive detailed data, we could not apply a qualitative content analysis on the transcribed interview section of their knowledge and attitudes. Instead, we summarize the results in this part to give a brief overview. During the focus group interviews, the students’ responses repeatedly revealed their limited prior knowledge of this topic. When the students were asked what they understood by animal testing, it became apparent that it was not clear to the students’ which criteria must be fulfilled to speak of animal experiments. For example, observations and behavioral experiments were also counted as animal experiments, respectively, and it was obviously not clear to some students if they count them as animal experiments. Students also missed a sense for the legal aspects in the development of new medicaments as they pointed out, for example, that humans could also step in as propositi in experiments instead of animals. Animal testing, however, is mandatory in the preclinical phase of drug development before a medication is tested on humans in the clinical phase. Even in this initial phase of the focus groups, the students kept saying that animal testing is fine as long as the animals do not have to suffer. All groups mentioned medical research and testing for cosmetics as examples of the use of animal testing. Interestingly, some students immediately revealed that they are less opposed to animal experimentation for “*useful areas*” like medical research instead of cosmetics, which they described as unnecessary.

### 3.2. Criteria Adolescents Used for the Evaluation of Animal Experimentation

In the qualitative content analysis, we found 19 subcategories which we structured into six main categories (Table 1). More than 60% of the evaluations fell into four subcategories (each of the subcategories reached an amount over 12%): *high relevance of research, the research is not relevant, animals suffer,* and *alternative methods possible*. Those four subcategories were present in all the focus group discussions and can thus be considered as particularly important. Although the students revealed that they did not have any prior in-depth knowledge on the topic of animal experimentation, none of them had problems in evaluating the given examples and coming to a reasoned decision.

*Extent of relevance of research:* One main criterion (with 34.4% of the total percentage and being the most common main category) for the evaluation of the animal experimentations was the aspect of received benefits for humans. The adolescents reflected in their discussion on the usefulness of research. They considered, for example, if the experiment could prevent the death of many people, i.e., could “*save*” people who suffer from a disease or was generally useful for humans. Other considerations focused on the frequency of diseases in the population. This was especially relevant in the context of cancer research as the following statement shows: “*Cancer is simply a strong disease in our society. So that is widespread, and I think something should be done about it. And even if it’s not so good for the animals, but I just think it should be done.*” [FI28B1] (The code indicates the transcipt reference). In contrast, the students described some of the experiments as “*unnecessary*” or “*useless*”. This argumentation was often related to the existence of alternative methods and was mainly present for obesity and cosmetic research: “*I think it’s incredibly strange and absurd to make drugs against obesity, because then somehow society must make sure that it doesn’t happen. Excuse me for saying this, but I think it’s absurd to breed pigs to an extent when it is necessary to develop a drug against obesity.*” [FI16B4].

*Extent of animal suffering/death:* The extent of animal suffering or the possible death of the animals was the second most common main category (its frequency was 31.3%) and was present in the evaluation of all experiments. This was expectable as the description of the experiments included the extent of the animals’ suffering. The students frequently mentioned the possible death of the animals during or after the experiment when the description stated that there would be at least medium pain (in cancer and migraine research): “*With cancer it is clear that they die.*” [FI35B2]. Some participants were also aware that animal experiments, in general, often end in the death of the animals: “*Whereby they [the animals] will probably still not be released after the experiment, but euthanized.*” [FI16B3]. Animal suffering as well as the death of the animals was very often mentioned in the evaluation of the experiments. These aspects, however, played only a subordinate role in the decisions on the approval of the specific examples (see Table 2). One other important aspect for the evaluation of the animal experiments in the context of suffering was reciprocal fairness between humans and animals. The students described specific experiments as “*mean*” or “*unfair*” and criticize that a healthy animal was made sick: “*But I honestly think it’s a little mean that animals have to suffer just because people eat too unhealthily and don’t exercise.*” [FI47B4].

*Existence of alternative methods*: The students frequently reflected on possible alternatives. As already described above, experiments for which they saw possible alternatives were often judged unnecessary. Alternatives were mostly described for cosmetic research, as many products already exist, as well as for obesity research because the existence of diet, sports, or surgical options: “*But as a matter of fact, for most people it is really not necessary, because through normal exercise and change of diet also the weight can be regulated.*” [FI16B1]. Interestingly, the students mentioned in one third of the coded comments in the subcategory “alternative methods possible”, that humans could replace animals in the experiments. Students referred in their comments mainly (but not exclusively) to experiments which resulted in no pain to light pain: “*You can also give a very small amount to a person, so to speak, like a vaccination, to see what happens.*” [FI35B1]; “*If no pain is expected, then it could theoretically be done in humans.*” [FI47B3]; “*This can be tested on humans as well and antihistamine can be given if any allergic reaction develops.*” [FI16B3]. Although it was described in most experiments that one aim is to test for side effects, students did not mention this aspect when they considered humans as possible propositi.

*Conditions of the animal experiment:* The students, only to a limited extent, reflected on the conditions of the animal experiments. Exemplary comments in this context were the comparison of the treatment of the animals during the experiment (mainly in the case of obesity research) with “*normal*” animal treatment in well-known processes like farming: “*Pigs are fattened anyway, nothing changes.*” [FI35B2]. The discussions rarely touched on the possible side effects of the developed medicaments. However, students may have lacked an understanding of the extent to which unexpected side effects could be dangerous in the subsequent use of medications.

*Assessment of knowledge gain:* During the discussion, the participants sometimes examined and evaluated the scientific process as well as the potential increase in knowledge gained. A possible knowledge gain was evaluated positively although it was only pronounced in rare cases: “*In any case, the fact that it can bring great progress in cancer research speaks for itself.*” [FI16B1]. In all focus groups, it happened at least once that the students considered if the experiment or its methodology made sense. They questioned, for example, whether it is possible to know undoubtedly if a medicament against migraines could work, as the animals cannot express their pain or the absence of pain in words: “*I wonder if it really helps. Because the mouse can’t say it has a headache.*” [FI47B3] (Although the species was declared in the description, students called mice, rats, and the other way round.) In some cases, participants also captured the problem that the results of animal models are not always applicable to humans: “*What if the drug works in the rat but not in humans?*” [FI35B1].

*Other criteria:* Some further criteria did not fit in the already described main categories and are described in the following paragraph. The perceived blame of certain people for having a specific disease was sometimes used for the evaluation of the experiments (in most of the cases students referred to obesity research): “*There are some [people] who can’t help being overweight, they have some kind of illness. But there are also many who can do something about it. And I think there’s no need for medication, because it’s actually your own fault if you’re overweight.*” [FI28B2]. Some students mentioned aspects which could be interpreted as an awareness that decisions about animal experimentation are dependent on the very personal situation of a human being. Still, these students’ comments were only vague: “*However, if you had cancer, I’m sure you’d want good drugs too, right?*” [FI35B2]. An interesting aspect of the focus group discussions was that reflecting together sometimes brought students to very general ethical questions like the moral standing of different living beings or the weighing of suffering and death: “*One tortures animals and [there] is just always this contradiction, one makes just another living being ill, in order to save another living being.*” [FI35B5]; “*But it’s also a question of whether dying is so much worse than suffering.*” [FI35B5].

All experiments were approved at least once, and all of the groups accepted at least one experiment of the four examples (Table 2). The reasons for the approval or disapproval focused on the relevance of the research, possible alternative methods, animal suffering, and the extent of one’s own fault in an illness. The groups discussed in most cases human benefits and animal costs when they evaluated the different examples of animal experimentation. When asked for a final decision, they stopped weighing different interests and prioritized one particular interest—usually the human’s ones. Only one group (group 1) also based their decisions on animal suffering as the main criterion for their approval or disapproval. One of our groups (group 4) also accepted animal experimentation for cosmetic purpose and argued: “*with deodorants, it’s important that the side effects are tested, because you can’t just let a deodorant loose on mankind, let’s say, because otherwise many allergies could arise.*” [FI28B4]. The same group used the extent of a patient’s fault for getting a disease as a decision aid. They tendentially approved of migraine research as people don’t have any blame getting migraine. Obesity research was tendentially not approved as they saw obesity as self-imposed.

### 3.3. Adolescents’ Reflections on the Prohibition of Animal Experimentation

At the end of the focus group discussions, the facilitator asked the participants how they assessed a complete ban of animal experimentation. Summarizing the results, all groups mentioned reasons against a complete prohibition, and all of them excluded at least cancer research from such a complete ban. The reasons against a complete prohibition were manifold. The students mentioned that it would be more difficult to develop drugs further and there would be a reduction in the offering of medicaments. They also described possible negative effects like a stagnation in cancer research and that the aim of healing cancer would recede into the distance. Some students had the fear that possibly humans would step in as propositi and be exposed to the risk of the side effects. One group also expressed concern that a complete ban would have consequences (at least in other countries) that would be even more morally reprehensible, such as trying to recruit vulnerable groups as subjects for experiments (e.g., homeless people).

B1 Or [the experiments would be] relocated to states where it’s still allowed (Abbreviations: F—Facilitator, B1–B5—number of the participants).
B4 Exactly, or simply some people who don’t have so much to say are taken and forced to serve for it now.

[...]

B3 That it is offered to homeless people or so.
B2 Or just, as we said, to people, to any homeless people, and if it’s the last thing they [the pharmaceutical industry] can do.
F Okay.
B1 Or you just promise him money for it.
B2 And yeah, I mean, I think in general, too, that the pharmaceutical industry could do well with that. Because they’re all a bit unscrupulous after all.

[...]

B5 I think that Asia, that is, many Asian countries—China, Japan and possibly also Korea, Thailand and so on—have a completely different view of human life.
B4 Yeah. Yes.
B5 Because with us [in our country], the individual has much more value than in these countries, so that has simply developed culturally. And I think that human experiments would then also gain in popularity. [FI16].

Discussing the possibility of using people instead of animals, the participants also came back to animal ethics issues as the following statement shows: “*People can choose for themselves whether they want to test it [a medicament], and the animals, they are simply taken.*” [FI47B3].

## 4. Discussion

The results indicate that adolescents can discuss the topic of animal experimentation quite similar to adults; in the present study, they used almost identical criteria to evaluate specific animal testing scenarios like adults did in previous research [11]. The participants came to decisions on the topic of animal experimentation although they considered their own knowledge about the whole topic as low.

All groups approved and disapproved of different scenarios of animal experimentation. This indicates that the groups weighted the criteria anew for each experiment. According to our interpretation, instead of a general approval or rejection of animal experiments, the participants tended to make a specific assessment in view of a concrete scenario. Individuals who have these “highly flexible attitudes” are referred to as “reserved” by Lund et al. [11], and we follow this categorization.

A wide range of criteria were used by the participants to evaluate each animal experiment, with considerations often revolving around the relevance of research, possible alternatives, and animal costs. In the prior discussions of the focus groups, the process of considering human benefits and animal costs was present, which has already been described in previous research [11,21]. However, in the final decision, the participants did not refer to many different criteria, instead each group focused on two main ideas (which differed between the groups) for all examples of animal experimentation. First, all groups used the “extent of relevance of research” as one main idea. The second main idea was in two groups the “existence of alternatives”, in one group the “level of animal suffering”, and in one group the “extent of fault”. Interestingly, it was not always possible to anticipate the final decision based on the comments the students made in the prior discussion. Group 3, for example, talked much more about animal costs (suffering, death, long term effects) than about human benefits when they discussed cancer research. In their decision, however, they did not refer to animal costs once; instead, it was immediately clear for the group that the human benefits are predominant.

This result raises the general question of how individual people arrive at a judgment. An explanation can possibly be found in Haidt’s social-intuitionist model. He describes intuitive processes, social interactions, and cultural backgrounds as part of a moral judgment formation. More precisely, he assumes that people make their moral judgments based on the intuitions they have in a specific situation. This process happens quickly and automatically and is effortless for the person. According to Haidt, the judgment is only justified afterwards and *only* if the person is asked to do so (as we did in our focus group interviews), because this process is strenuous [22].

Furthermore, people may be influenced in their decision by the arguments they hear from their interlocutors or by the personal closeness they have to certain people in the group, and social pressure may play a role too [22]. The latter aspect is especially relevant in the school environment, since the class community can be understood as a social system in which there are several small groupings that are close to each other in different ways. It is therefore possible that the group (which knew each other quiet well) tried to focus on reasons which had shown to be acceptable for most group members in the prior discussion. As the decision took place after the discussion of all experiments it is also possible that students were already a bit tired at the end of the interview and did not want to consider their reasons again. This idea is based on the observation that sometimes students just tried to make a ballot to come to a fast decision and delivered their reasons only after they have been asked to by the facilitator.

As we mentioned earlier, participants described their knowledge of animal experimentation as low. It is possible that this low level had an impact on the assessment. For example, the question arises: to what extent did participants have an understanding of the pain levels described? This is anything but a trivial question, as it touches on the fundamental issue of the extent to which we can understand and empathize with another creature’s pain. This question concerns scientists as well as young people. We did not explore the participants’ understanding of each experiment in this study. However, this could be an interesting question for further research: to what extent does the understanding of animal experiments have an influence on their evaluation? In this context, it would also be interesting to investigate what effect the use of images can have on the comprehension and evaluation process. On the one hand, images could contribute to a better understanding of how animal experiments take place; on the other hand, pictures are always pictures of a certain situation. This can lead to discussing details of this situation rather than the general subject matter. These issues would need to be explored in future research projects.

A key finding of the survey can be summarized as follows: The adolescents in the study had considerably more positive attitudes toward animal experimentation than might have been expected based on the literature, in which young people tended to be described as more critical than older people [9,10,13,23]. All groups identified positive aspects of animal experimentation and accepted at least one scenario of animal experimentation. Most of the groups rated half of the examples presented as acceptable. We did not have a group or a participant that was exclusively against animal testing.

There are at least four possible explanations for the finding of a considerably positive attitude toward animal experimentation. (1) One reason could be found in the area of the survey: Although Austria is on the second to last position in the acceptance of animal experimentation compared to other European countries, [23] which speaks against an expectable high acceptance, previous studies indicated that young people in Austria are more likely to support animal experimentation than older people [23,24]. Future research would have to clarify whether this generation gap is significant and what the reasons for it may be. (2) People are often generally described as “approver”, “disapprover”, or “reserved” based on their tendency to evaluate animal experimentation [11,21]. Although “reserved” people—who form their position on animal experimentation anew in each scenario—are dominant at least in the Danish population according to Lund et al., approximately 50% also belong to the other two types [21]. One explanation for our participants acting mostly reserved may be the already described social pressure which possibly exists in groups of school classes. A reversed position may be easier manageable for group decisions. Although the students were told at the beginning of the interview that there were no wrong views, the need to act uniformly with the group could still have prevailed. Furthermore, to ensure that the participants reflect on their own opinions, they were first asked to write down their own assessment of the animal experiments prior to the discussion. All students presented their own opinions in the group discussion without hesitation. Nevertheless, a certain degree of the so called “bandwagon effect” cannot be completely ruled out. Even if it was hard for us to observe, it is known from the literature that students act in groups according to specific social norms. One student often takes the role of a leader who clearly influences the group’s actions [25,26]. Another explanation for the relevance of the reserved position could be the tendentially scientific description of the scenarios. Laslo and Baram-Tsabari could show that the ethical or scientific framing of articles influences the use of supporting or opposing comments. In their study, scientifically framed newspaper articles on animal experimentation were followed by both supporting and opposing comments, whereas, ethically framed articles were followed by more opposing comments [27]. Although we did not use a whole article, this aspect may have played a role.

(3) Most of the scenarios we used focused on medical research, which is the most accepted area of animal experimentation [10,13,28,29]. Additionally, no species was used in the scenarios which is known to be less accepted (like dogs or non-human primates) [28]. Instead, most scenarios stated the use of rodents which are generally the most accepted group for animal experimentation, according to previous surveys [9,10]. (4) Finally, the qualitative research method we used may have played a central role on the outcome. It is already known that general questions on the approval of animal experiments can have a different outcome than judging specific examples. Furthermore, previous studies have mainly explored attitudes towards animal experimentation using quantitative methods like questionnaires with rating scales, a form of answer that does not allow a more precise consideration of the participants [10]. Our participants, in turn, had a much longer time to think about the pro and contra arguments in the discussions and gained new perspectives by other participants. As we did not collect their prior attitude on animal experimentation before the focus group, there is even the possibility that they might have changed their minds during the discussion.

The question, therefore, arises: to what extent it is possible to understand the attitudes of specific groups towards animal experiments solely on the basis of questionnaires? It is likely that one and the same person would answer general questions about animal experiments in questionnaires differently than they would evaluate specific examples in discussions. Reflections on specific scenarios of animal testing in discussions could, for example, promote a change of perspective to the people who need this medication. There is no question that both qualitative and quantitative research methods have their advantages as well as disadvantages. This is not the place to discuss these strengths and weaknesses in their entirety. Roughly speaking, qualitative methods are not about discovering numerical correlations or representative statements, but—in this context—about illuminating how young people think and talk about animal experiments. What wording do they use? What themes emerge? How do the debates develop? However, due to conducting the work with small samples, the results cannot be generalized (this limitation also applies to the study presented here). Quantitative methods can, with well-developed and accurate instruments, lead to results that can be representative of the population with the right sample selection [30]. More research is necessary to analyze different outcomes in the context of animal testing based on the research method.

Some responses of the participants indicated that they are less concerned about the practices regarding animal usage that they are quite familiar with, e.g., livestock farming. For example, they compared the treatment of the animals during the experiment (mainly in the case of obesity research) with “normal” animal treatment in well-known processes like farming, justifying the animal experimentation: “*Pigs are fattened anyway, nothing changes*.”

It is known that laypersons are largely unfamiliar with animal experimentation [11] and their knowledge level is generally low [8]. It therefore seems necessary to make animal experiments better known to the general public in order to reduce the distance between scientists and laypeople. A more open communication of the performing institutions as well as a simplified presentation of the research for the public are seen as necessary criteria [4]. The results of the current study also show that the understanding of drug development, in general, needs to improve. Students’ ideas that humans could replace animal testing as test subjects show the clear gaps in their knowledge: “*If no pain is expected, then it could theoretically be done in humans.*” Animal testing is a legally required phase in drug development [31] and this should be a known aspect in society.

The participants frequently reflected on the possible “alternatives”. At first glance, this fits the topic; after all, alternatives play an essential role in the context of the “3Rs”. However, the participants focused on other alternatives; they are not concerned with other paths within research, but with alternatives to problem solving outside of science, in general. Their “replacement”, therefore, concerns the way of life, not science. For example, they asked: can the problem that is to be solved with animal experiments not be avoided in any other way? This is most obvious when it comes to the question of obesity; can’t it be reduced by other means? These questions lead, as we will discuss below, to the question of “guilt”.

Generally, we hypothesize that the focus group discussions took place largely within the framework of anthropocentric ethics. Pathocentric considerations such as “animals should suffer as little as possible” did play a role, but as soon as the human benefit seemed great enough, the animals’ interests were put aside. Accordingly, the debates did not focus on the situation of the animals, but they addressed a variety of frameworks and issues beyond the situation in the laboratory. For instance, one aspect that has so far played a comparatively minor role in the scientific debate surrounding the acceptance of animal experiments, but which is certainly an interesting finding of the present study, is the question of one’s own fault for a disease. Animal experiments that seek therapies for diseases for which the affected people themselves are “partly to blame” were judged more critically than others. The animal experimentation debate can thus be intertwined with the concept of “guilt” and accompanying questions. This aspect exemplifies that the evaluation is not based solely on the situation of the animals, but also makes quite different conditions an issue. It is a well-known phenomenon that people have more negative attitudes towards overweight people if they consider weight to be a controllable factor [32,33]. This aspect might have been relevant for the evaluation of the experiment. The question arises whether the aspect of guilt is, generally, a factor that plays a role in the evaluation of animal experiments. Further studies are necessary to take a closer look at this aspect and to analyze the relevance of the criterion for decisions.

## 5. Conclusions

The aim of this study was to gain a deeper understanding of the ethical considerations of young people regarding animal experimentation. Adolescents evaluated animal experiments with similar criteria to adults and reshaped their position with each animal experiment. Students focused mainly on the following criteria: the relevance of research, the extent of animal suffering, and the existence of alternatives. Participants’ decisions showed clear characteristics of the use of anthropocentric ethics. Animal interests received little consideration in decisions to approve animal experiments once the human benefits seemed great enough. Contrary to the existing literature (which is mainly based on questionnaires), relatively positive attitudes toward animal experimentation were found in our respondents. A significant reason for this result could be the applied qualitative analysis of attitudes. In general, we suggest the use of more qualitative studies to analyze attitudes of the public as the outcome of studies which focus on in-depth analyzes of attitudes are considerably different compared to those conducted using quantitative research.

## Figures and Tables

**Table 1:** Overview of the criteria the students used to evaluate animal experimentation. The data is presented with the percentage of main category and subcategory frequency of the qualitative content analysis. IRR Krippendorff’s Alpha = 0.81.

Main Category and Total Percentage	Subcategory	Percentage of Subcategory
Extent of relevance of research—34.4%	High relevance of research	18.0%
	Research is not relevant	16.4%
Extent of animal suffering/death—31.3%	Animals suffer	15.6%
	Animals die	8.6%
	Animals do not suffer	4.3%
	Animals do not die	2.3%
	Death is natural	0.4%
Existence of alternative methods—13.7%	Alternative methods possible	12.5%
	No alternatives	1.2%
Conditions of the animal experiment—9.4%	Number of animals used	2.7%
	“Normal” animal treatment	2.3%
	Long term effect for the animal	2.0%
	Side effects not clear	1.6%
	Animal husbandry	0.8%
Assessment of knowledge gain—5.1%	Doubts about the sense/methodology of the experiment	3.5%
	New knowledge is generated	1.6%
Other criteria—6.3%	Extent of fault	3.1%
	Depends on the moral status of the living being	2.0%
	Decision is situational	1.2%

**Table 2:** The table shows the opinions and main reasons used for the decisions for all four focus groups. (+) approved, (+) tendentially approved, (−) tendentially not approved, − not approved.

Experiment	Group 1	Group 2	Group 3	Group 4
Cancer research	− Animals suffer	+ High relevance of research	+ High relevance of research	+ High relevance of research
Migraine research	− Animals suffer	+ High relevance of research	+ High relevance of research	(+) High relevance of research, Extent of fault
Obesity research	+ High relevance of research, Animals do not suffer	− Alternative methods possible, Research is not relevant	− Alternative methods possible, Research is not relevant	(−) Extent of fault
Cosmetics research	− Research is not relevant	− Alternative methods possible, Research is not relevant	− Alternative methods possible, Research is not relevant	+ High relevance of research

## Data Availability

The data presented in this study are available on reasonable request from the corresponding author taking into account the applicable data protection law such as the specific rules of the consent authorization of the participants.
